# Deep learning-based automatic scoring of drug-induced sleep endoscopy in obstructive sleep apnea

**DOI:** 10.1038/s41746-026-02673-8

**Published:** 2026-04-28

**Authors:** Jin Youp Kim, Sue Jean Mun, Young Seo Baik, Young Seop Lee, Young Jae Kim, Jayoung Oh, Gwanghui Ryu, Chung-Man Sung, Sung Jae Heo, Hyung Chae Yang, Hyun Jik Kim, Hyo Yeol Kim, Kyu-Sup Cho, Kwang Gi Kim, Chae-Seo Rhee

**Affiliations:** 1 Department of Otorhinolaryngology–Head and Neck Surgery, Ilsan Hospital, Dongguk University, Goyang, Gyeonggi-do Republic of Korea; 2 Department of Otorhinolaryngology–Head and Neck Surgery, Pusan National University Yangsan Hospital, Yangsan, Gyeongsangnam-do Republic of Korea; 3 Department of Biomedical Engineering, Gachon University, Seongnam-si, Gyeonggi-do Republic of Korea; 4 Medical Devices R&D Center, Gil Medical Center, Incheon, Republic of Korea; 5 Department of Gachon Biomedical & Convergence Institute, Gachon University Gil Medical Center, Incheon, Republic of Korea; 6 Department of Otorhinolaryngology–Head and Neck Surgery, Seoul National University Bundang Hospital, Seongnam-si, Gyeonggi-do Republic of Korea; 7 Department of Otorhinolaryngology–Head and Neck Surgery, Samsung Medical Center, Sungkyunkwan University School of Medicine, Seoul, Republic of Korea; 8 Department of Otorhinolaryngology–Head and Neck Surgery, Chonnam National University Medical School, Gwangju, Republic of Korea; 9 Department of Otorhinolaryngology–Head and Neck Surgery, School of Medicine, Kyungpook National University, Daegu, Republic of Korea; 10 Department of Otorhinolaryngology-Head and Neck Surgery, Seoul National University College of Medicine, Seoul, Republic of Korea; 11 Department of Otorhinolaryngology–Head and Neck Surgery, Pusan National University School of Medicine, Pusan National University Hospital, Busan, South Korea; 12 Department of Biomedical Engineering, College of IT Convergence, Gachon University, Seongnam, Gyeonggi-do Republic of Korea

**Keywords:** Laboratory techniques and procedures, Endoscopy

## Abstract

Polysomnography is the standard tool for assessing obstructive sleep apnea (OSA) severity; however, it does not provide information regarding the anatomical site or extent of upper airway obstruction. Drug-induced sleep endoscopy (DISE) serves as a dynamic method to evaluate airway collapse under sleep-like conditions, thereby helping to bridge this gap. However, its clinical utility is limited by inter-observer variability and subjectivity in interpretation. We developed internally and externally validated deep learning models utilizing convolutional neural networks based on EfficientNet-B2 and Attention Multiple Instance Learning to predict the degree of airway obstruction (DISE-V-obs, DISE-OTE-obs) and the primary cause of obstruction (DISE-OTE-cause) using DISE videos from 1904 patients across five Korean hospitals. The F1 scores for DISE-V-obs, DISE-OTE-obs, and DISE-OTE-cause were 84.7%, 74.7%, and 88.2%, respectively. These objective predictions of obstruction degree and primary cause may enhance clinical decision-making and treatment planning for patients with OSA.

## Introduction

Obstructive sleep apnea (OSA) is characterized by repeated episodes of upper airway collapse during sleep, often accompanied by arousals, with or without oxygen desaturation^1^. It negatively affects quality of life by causing daytime sleepiness and impaired cognitive function and is associated with numerous long-term comorbidities. Cardiovascular conditions such as hypertension, myocardial infarction, arrhythmia, stroke, coronary artery disease, and congestive heart failure—as well as neurocognitive disorders including dementia and Alzheimer’s disease—are well-established comorbidities of OSA^2–4^. Polysomnography (PSG) is the gold standard for evaluating OSA. The apnea–hypopnea index (AHI) quantifies the average number of respiratory events, including obstructive apneas and hypopneas, per hour of sleep^5^. Although PSG determines OSA severity, it does not provide information about the site or degree of airway obstruction. Identifying the site and degree of obstruction is crucial for selecting appropriate treatment options, such as positive airway pressure, surgery, or oral appliance therapy. Drug-induced sleep endoscopy (DISE) is commonly used to assess the anatomical characteristics of airway obstruction. DISE allows dynamic visualization of the upper airway during a pharmacologically induced sleep state^6^. Unlike PSG, which reflects the physiological consequences of respiratory compromise, DISE offers real-time visualization of obstruction sites. When used in conjunction with PSG, DISE helps identify both anatomical and non-anatomical pathophysiological mechanisms underlying OSA in individual patients, thereby facilitating personalized treatment planning^7^. DISE has also demonstrated utility in predicting the success of mandibular advancement devices^8^, positional therapy^9^, and various surgical interventions for OSA^10,11^. Additionally, it can predict poor outcomes after upper airway stimulation in patients with specific obstruction patterns^12^.

During DISE, an endoscope is inserted through the nasal cavity to assess the upper airway from the nares to the glottis. Following the procedure, the surgeon applies the VOTE classification system—comprising the velum, oropharynx lateral walls, tongue base, and epiglottis—a widely accepted scoring method used to identify the sites of collapse within the upper airway and evaluate the degree of obstruction. The endoscopic examination is typically performed in two regions: the velum and the OTE (oropharynx lateral walls, tongue base, and epiglottis). Physicians score DISE results based on two video recordings—one each for the velum and OTE—that capture upper airway movements. However, several challenges are associated with DISE scoring. Because the interpretation of three-dimensional movement relies on two-dimensional imaging, variations in scoring may occur depending on the rater’s level of experience. Accordingly, substantial variability in DISE scoring was reported in previous studies^11,13–15^. This inter-rater variability is clinically concerning because it may lead to inconsistent or inappropriate management decisions. Although no study has explicitly quantified the rate of misdiagnosis or unnecessary surgeries caused by low agreement in VOTE scoring, unreliable DISE interpretation could contribute to such issues. Notably, DISE findings have been shown to change the surgical treatment plan in ~43–78% of cases^6,7^. These findings indicate that if DISE interpretation is inaccurate or inconsistent among clinicians, it may lead to divergent treatment strategies, potentially resulting in additional, alternative, or even unnecessary procedures. This is particularly relevant given that surgical treatments target different anatomical structures—genioglossus advancement and tongue-based radiofrequency target the tongue base, whereas palatopharyngoplasty predominantly addresses the lateral pharyngeal walls^14,16^. Therefore, inaccurate identification of the site of obstruction could result in surgical interventions that fail to resolve the primary airway collapse, leading to unwarranted procedures. Therefore, enhancing the objectivity and inter-rater agreement of DISE scoring systems may be critical for improving treatment accuracy and clinical outcomes.

In recent years, deep learning has achieved remarkable breakthroughs, establishing itself as a transformative technology across diverse scientific and industrial domains. By leveraging high computational power and large datasets, deep learning enables the analysis of complex patterns and supports predictive tasks. These capabilities may be applied to automatically score DISE results and reduce inter-rater variability. A study by Hanif et al. ResNet-18 and a bidirectional long short-term memory-based neural network were employed to automatically score the degree of obstruction in DISE videos. The deep learning model achieved a mean F1 score of 70% from 281 DISE videos; however, external validation was not performed, and the dataset was relatively small^17^.

This study aimed to develop an internally and externally validated automatic DISE scoring system to predict the degree and cause of upper airway obstruction. To achieve this, a convolutional neural network (CNN)-based obstruction prediction algorithm was developed using DISE videos collected from multiple hospitals.

## Results

### Clinical characteristics

**Fig. 1:**
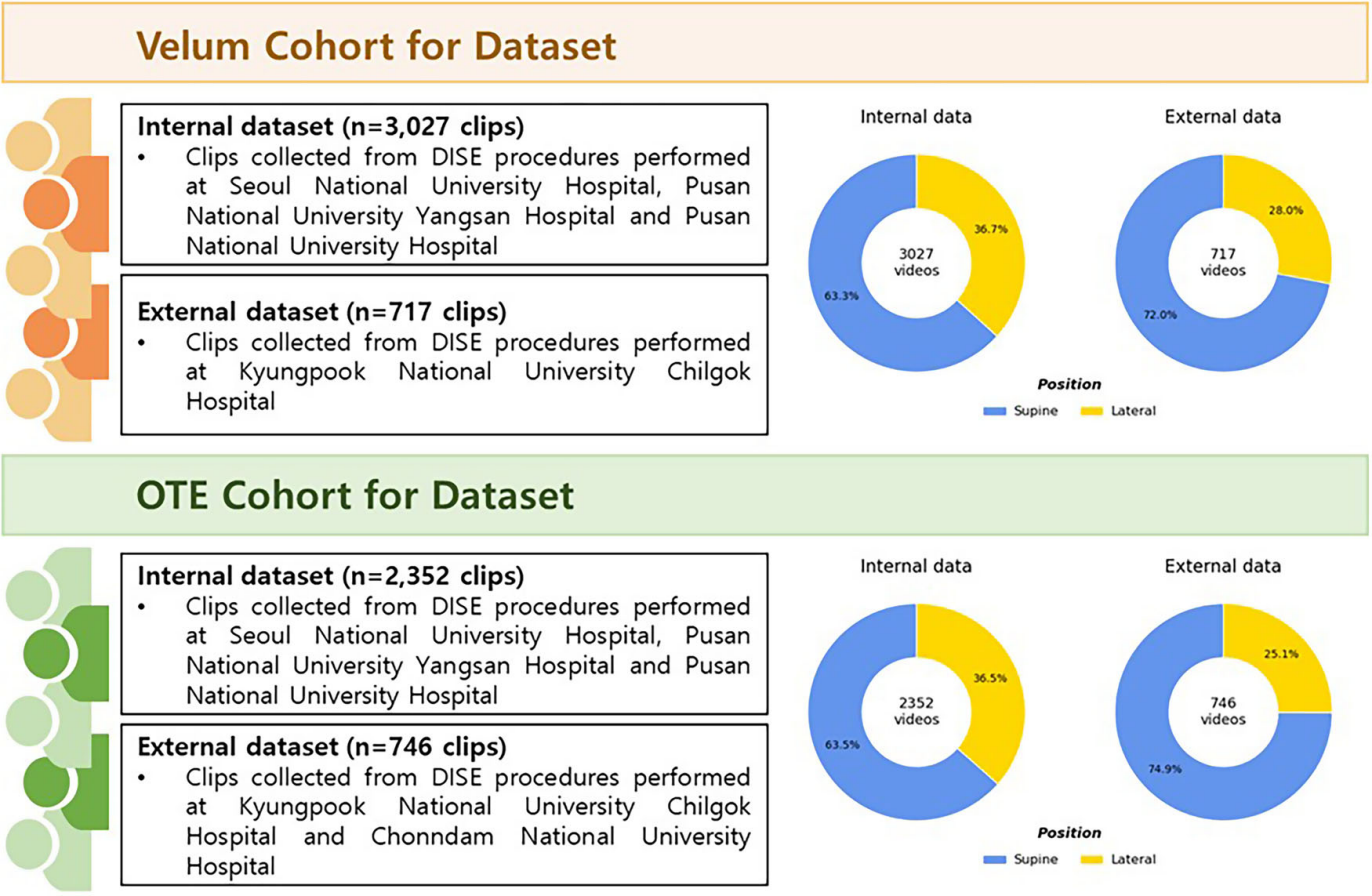
Overview of the study cohort across five participating institutions. The diagram summarizes distribution of video clips for the Velum and OTE regions used in model training and validation.

A total of 1904 patients were enrolled in the study. The demographics and clinical characteristics of the DISE video data (velum and OTE) are presented in Table 1. The study population included 150 patients with snoring and 1754 patients with OSA. The mean (SD) age was 46.07 ± 14.0 years, and the population was predominantly male. The mean SpO2 recorded during PSG was 91.8 ± 5.3. An overview of the dataset composition and positional distribution of the DISE videos is illustrated in Fig. 1. The Velum cohort comprised 3744 video clips: 3027 from the internal dataset and 717 from the external dataset. The OTE cohort consisted of 3098 videos, with 2352 from the internal dataset and 746 from the external dataset. Across both cohorts, most videos were recorded in the supine position. Specifically, the supine position accounted for 63.3% and 72.0% of videos in the Velum internal and external datasets, respectively, and for 63.5% and 74.9% in the OTE internal and external datasets, respectively.

**Table 1:** Demographic and clinical characteristics of patients in Velum and OTE datasets

Variables	Velum		OTE	
	Internal dataset (*N* = 3027)	External dataset (*N* = 717)	Internal dataset (*N* = 2352)	External dataset (*N* = 746)
Age in years (mean ± SD)	46.0 ± 13.8	46.4 ± 14.7	45.9 ± 13.8	46.6 ± 14.8
Sex (M:F)	2522:505	573:144	1943:409	600:146
Obstruction (none:partial :complete)	593:718:1716	159:186: 372	1205:616:531	137:296:313
BMI (kg/m2, mean ± SD)	26.4 ± 4.0	26.0 ± 3.6	26.0 ± 5.2	25.8 ± 3.6
Position (supine: lateral)	1916:1111	516:201	1493:859	559:187
SpO2 during PSG (mean ± SD)	91.9 ± 5.4	91.1 ± 5.3	92.2 ± 5.0	91.1 ± 5.3
AHI (mean ± SD)	33.9 ± 25.5	30.7 ± 23.0	33.5 ± 25.1	29.8 ± 22.7

*AHI* Apnea–Hypopnea Index, *BMI* body mass index, *SpO₂* peripheral capillary oxygen saturation, *Velum* uvula, soft palate, and lateral/posterior pharyngeal wall, *OTE* oropharynx, tongue base, and epiglottis.

Supine and lateral positions indicate body posture during DISE evaluation. Internal and external datasets were collected from separate institutions as part of multicenter data acquisition.

### Performance of the DISE-V-obs model for velum obstruction degree classification

**Fig. 2:**
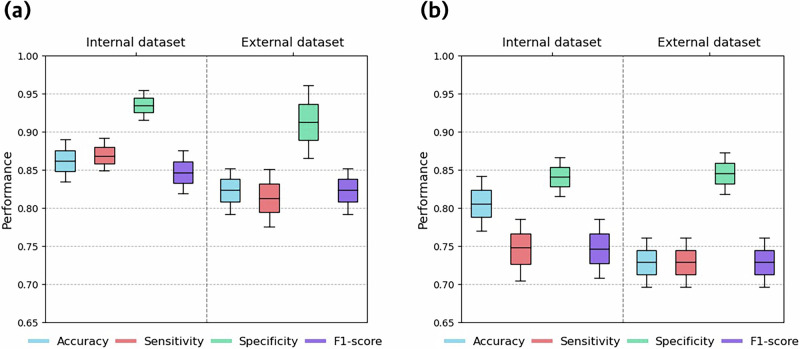
Performance of the DISE-V-obs and DISE-OTE-obs models in predicting obstruction degree. **a** Performance of the DISE-V-obs model for predicting the obstruction degree of the velum region. **b** Performance of the DISE-OTE-obs model for predicting the obstruction degree of the OTE region. Metrics include accuracy, sensitivity, specificity, and F1 score, each evaluated using fivefold cross-validation on both internal and external datasets.

**Fig. 3:**
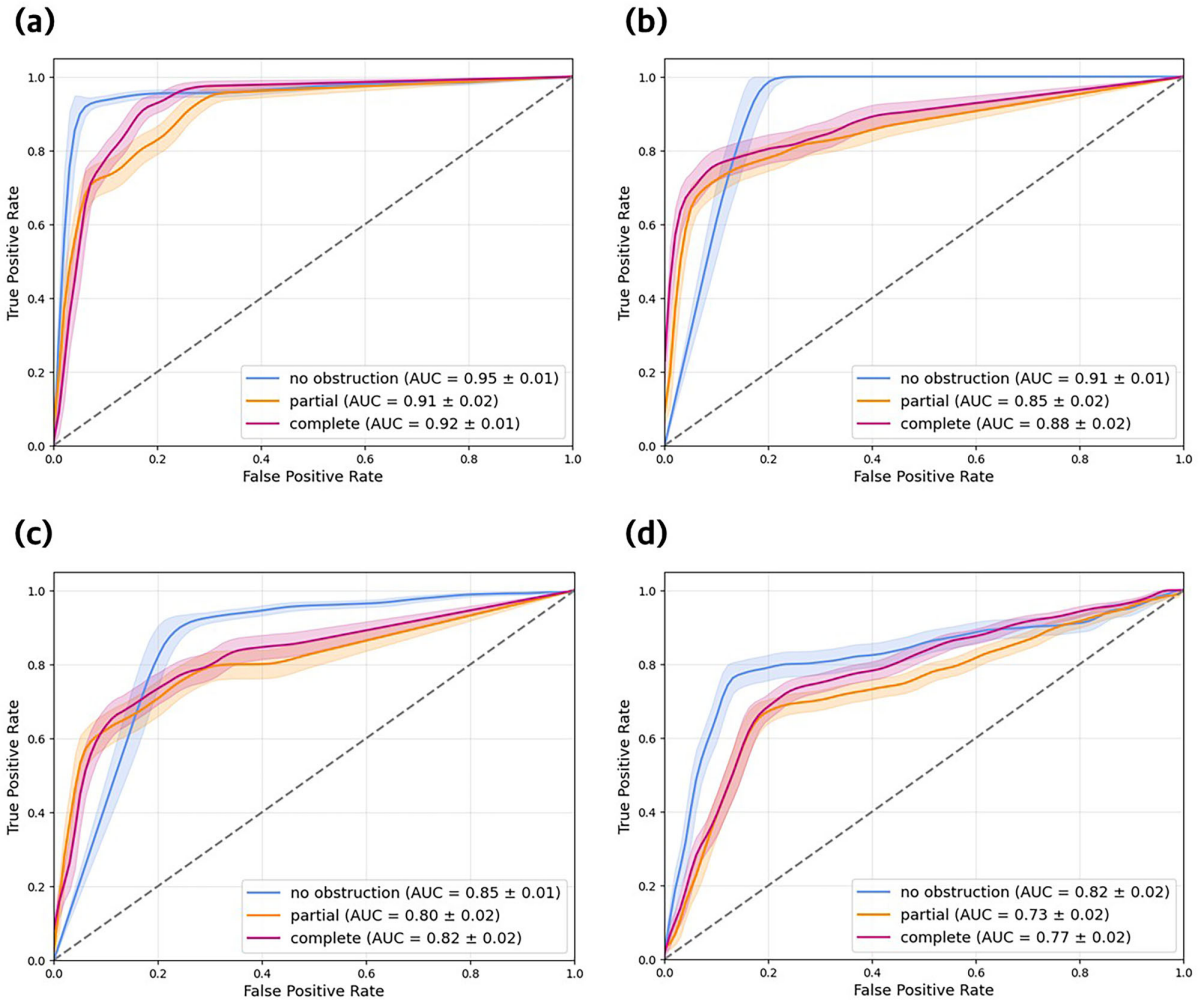
Receiver operating characteristic (ROC) curves for obstruction degree classification in the velum and OTE regions. ROC curves are shown for each obstruction class (no, partial, complete), based on internal and external validation datasets. **a** Velum internal dataset, **b** Velum external dataset, **c** OTE internal dataset, **d** OTE external dataset.

The degree of obstruction in the velum region during DISE was assessed using the deep learning model DISE-V-obs, which classified obstruction into three categories: no obstruction, partial obstruction, and complete obstruction. Model performance was evaluated on both internal and external datasets. DISE-V-obs achieved an average accuracy of 86.2% (95% CI: 83.5–89.0), sensitivity of 86.8% (95% CI: 84.9–89.2), specificity of 93.5% (95% CI: 91.6–95.5), and F1 score of 84.7% (95% CI: 81.9–87.6) on the internal dataset, as shown in the performance boxplot (Fig. 2). Performance was slightly lower on the external dataset, with an accuracy of 82.4% (95% CI: 79.2–85.2), sensitivity of 81.3% (95% CI: 77.6–85.1), specificity of 91.3% (95% CI: 86.6–96.1), and F1 score of 82.4% (95% CI: 79.2–85.2). Cohen’s kappa was 77.9% (95% CI: 73.4–82.1) and 71.5% (95% CI: 66.8–76.0) for internal and external datasets, respectively. The receiver operating characteristic (ROC) curve of DISE-V-obs (Fig. 3) indicated that model performance in predicting ‘no obstruction’ cases was highest, with an area under the receiver operating characteristic curve (AUROC) of 0.95 ± 0.01 in the internal dataset, followed by ‘complete obstruction’ (0.92 ± 0.01) and ‘partial obstruction’ (0.91 ± 0.02). In the external dataset, AUROC values were slightly lower: 0.91 ± 0.01 for ‘no obstruction’, 0.85 ± 0.02 for ‘partial obstruction’, and 0.88 ± 0.02 for ‘complete obstruction’. These findings suggest that the model performed well in distinguishing ‘no obstruction’, but the overlapping characteristics of partial and complete obstruction posed challenges for clear differentiation. In contrast, per-class F1-scores showed a different pattern (Supplementary Table 1), with ‘complete obstruction’ achieving the highest F1 score in both datasets (internal: 90.6%, external: 86.1%), followed by ‘partial obstruction’ (internal: 81.9%, external: 80.0%). Notably, the F1 score for ‘no obstruction’ was lower than its AUROC (internal: 81.9% vs. 0.95; external: 77.1% vs. 0.91), primarily due to classification challenges at the decision threshold as revealed by the confusion matrices (Supplementary Fig. 1). These discrepancies likely reflect class imbalance effects, where the underrepresented ‘no obstruction’ class faces difficulties in achieving optimal precision-recall balance.

### Performance of the DISE-OTE-obs model for OTE obstruction degree classification

The degree of obstruction in the OTE region during DISE was assessed using the deep learning model DISE-OTE-obs, which classified obstruction into three categories: no obstruction, partial obstruction, and complete obstruction. Model performance was evaluated on both internal and external datasets. DISE-OTE-obs achieved an average accuracy of 80.6% (95% CI: 77.0–84.2), sensitivity of 74.8% (95% CI: 70.5–78.6), specificity of 84.1% (95% CI: 81.6–86.7), and F1 score of 74.7% (95% CI: 70.8–78.6) on the internal dataset, as shown in the performance boxplot (Fig. 2). On the external dataset, performance was slightly lower, with an accuracy of 72.9% (95% CI: 69.7–76.1), sensitivity of 72.9% (95% CI: 69.7–76.1), specificity of 84.6% (95% CI: 81.8–87.3), and F1 score of 72.9% (95% CI: 69.7–76.1). Cohen’s kappa was 66.4% (95% CI: 60.4–72.3) and 58.0% (95% CI: 52.9–62.9) for internal and external datasets, respectively. The ROC curve of DISE-OTE-obs (Fig. 3) showed that the model performed best in predicting ‘no obstruction’ cases, with an AUROC of 0.85 ± 0.01 in the internal dataset, followed by ‘complete obstruction’ (0.82 ± 0.02) and ‘partial obstruction’ (0.80 ± 0.02). In the external dataset, AUROC values were slightly lower: 0.82 ± 0.02 for ‘no obstruction’, 0.73 ± 0.02 for ‘partial obstruction’, and 0.77 ± 0.02 for ‘complete obstruction’. These findings indicate that DISE-OTE-obs had difficulty accurately classifying partial obstruction due to its intermediate characteristics—similar to the challenges observed with DISE-V-obs. However, per-class F1-scores showed a different pattern (Supplementary Table 1). For the internal dataset, ‘no obstruction’ achieved the highest per-class F1 score (90.6%), followed by ‘partial obstruction’ (72.9%), and ‘complete obstruction’ (60.4%)—the latter showing a marked discrepancy from its AUROC. This divergence was due to classification errors between adjacent severity levels and class imbalance effects, as the less frequent ‘complete obstruction’ class faced challenges in achieving optimal precision-recall balance, as shown in the confusion matrices (Supplementary Fig. 1). Similarly, in the external dataset, the F1 score for ‘no obstruction’ (68.2%) was notably lower than its AUROC, reflecting similar challenges for this less frequent class at the decision threshold.

### Performance of the DISE-OTE-cause model for OTE obstruction cause classification

**Fig. 4:**
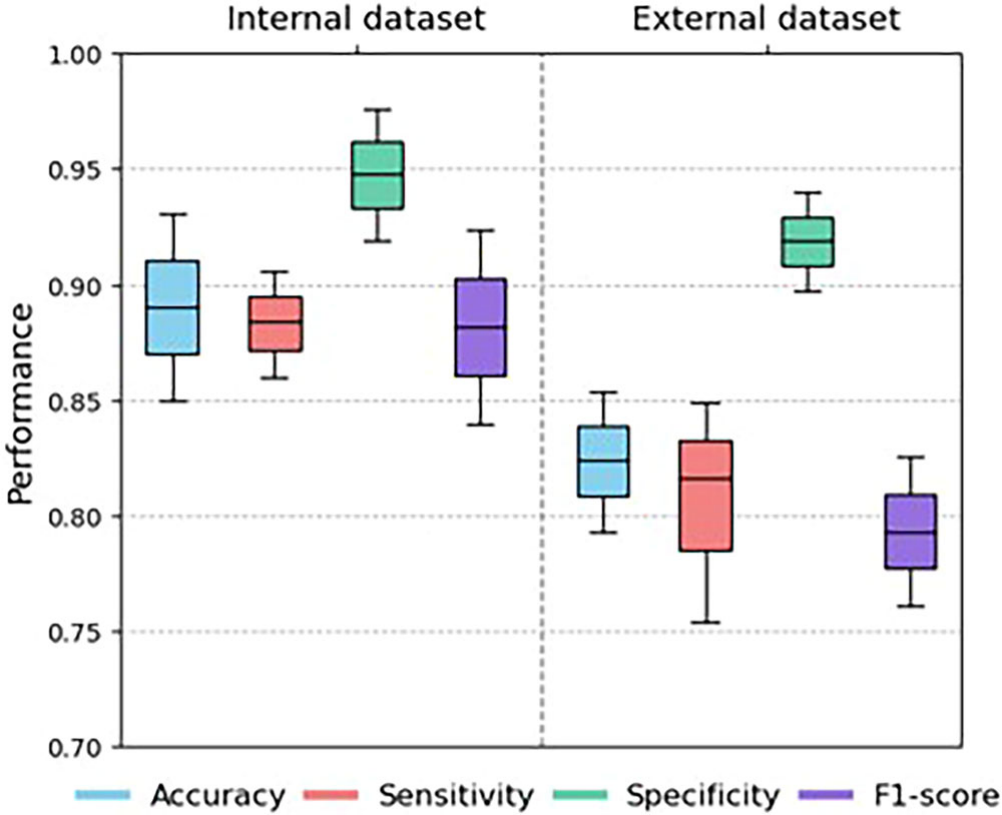
Performance of the DISE-OTE-cause model in predicting primary obstruction causes. Metrics include accuracy, sensitivity, specificity, and F1 score, evaluated using fivefold cross-validation on both internal and external datasets.

**Fig. 5:**
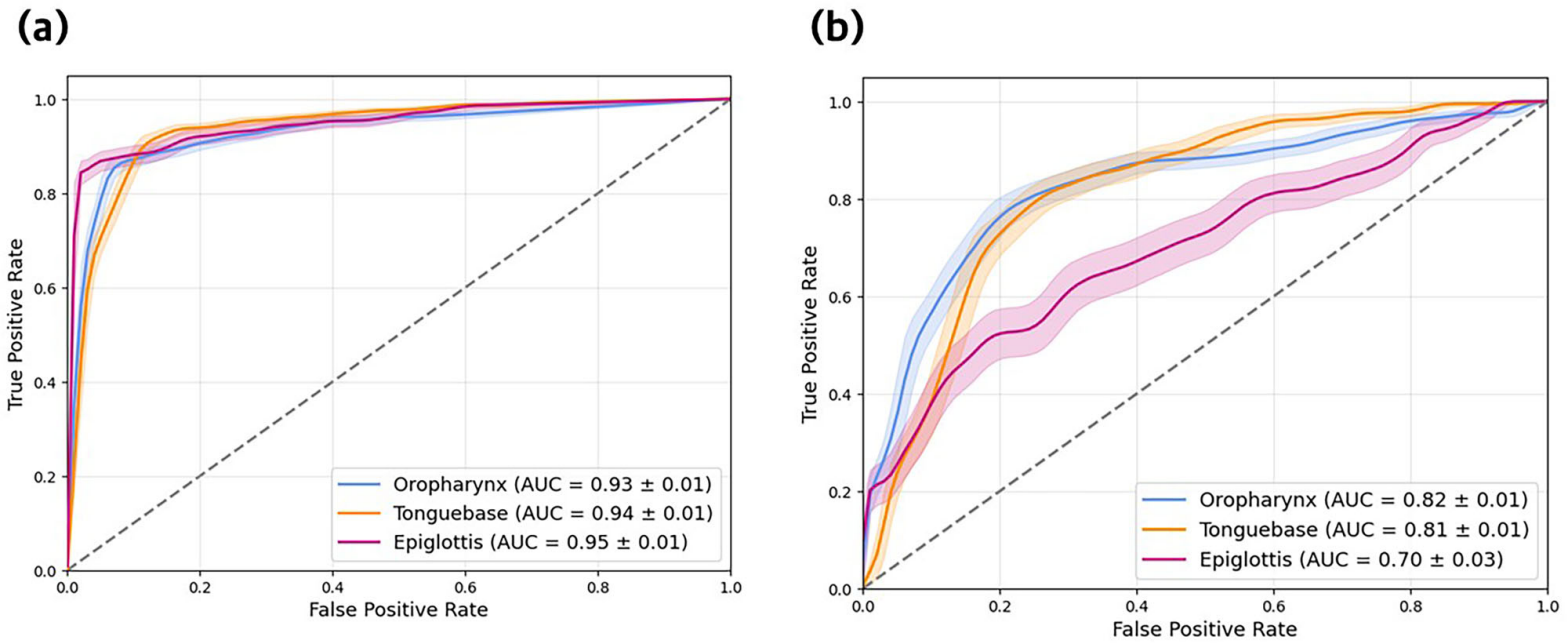
Receiver operating characteristic (ROC) curves for primary obstruction cause classification in the OTE regions. ROC curves are shown for each obstruction class (oropharynx, tongue base, epiglottis), based on internal and external validation datasets. **a** ROC curves for the internal dataset. **b** ROC curves for the external dataset.

The DISE-OTE-cause model demonstrated robust performance in predicting the primary cause of obstruction in the OTE region by classifying obstruction at the oropharynx lateral wall, tongue base, and epiglottis. On the internal dataset, the model achieved an average accuracy of 89.0% (95% CI: 85.0–93.1), sensitivity of 88.4% (95% CI: 86.0–90.6), specificity of 94.8% (95% CI: 91.9–97.6), and F1 score of 88.2% (95% CI: 84.0–92.4), as shown in the performance boxplots (Fig. 4). For the external dataset, performance was lower, with an average accuracy of 82.4% (95% CI: 79.3–85.4), sensitivity of 81.6% (95% CI: 75.4–84.9), specificity of 91.9% (95% CI: 89.7–94.0), and F1 score of 79.3% (95% CI: 76.1–82.6). Cohen’s kappa was 83.2% (95% CI: 76.2–88.6) and 71.9% (95% CI: 67.2–76.6) for internal and external datasets, respectively. The ROC curve (Fig. 5) indicated strong predictive performance for the internal dataset, with AUROC values of 0.95 ± 0.01 for epiglottis, 0.94 ± 0.01 for tongue base, and 0.93 ± 0.01 for oropharynx lateral wall. In the external dataset, AUROC values were 0.81 ± 0.01 for tongue base, 0.82 ± 0.01 for oropharynx lateral wall, and 0.70 ± 0.03 for epiglottis. These findings indicate relatively consistent performance in classifying oropharynx lateral wall and tongue base obstructions. However, challenges remain, particularly in classifying epiglottic obstruction in the external dataset. Frequent misclassifications of epiglottis obstruction—often labeled as tongue base—were observed in the confusion matrix (Supplementary Fig. 2). Per-class F1-scores showed generally good correspondence with AUROC values (Supplementary Table 2), though epiglottis in the internal dataset showed some discrepancy (AUROC: 0.95 vs. F1: 83.5%), likely due to its lower frequency. The external dataset maintained consistent alignment between discriminative and classification metrics across all classes.

### Interpretability assessment using grad-CAM

To enhance the interpretability and understand the spatial decision-making of our models, we employed Gradient-weighted Class Activation Mapping (Grad-CAM) on representative frames across various obstruction categories. Grad-CAM visualizations revealed that all models consistently focused on clinically meaningful anatomical regions, particularly the airway lumen and adjacent soft tissues, rather than on irrelevant artifacts such as saliva, glare, or lens distortion (Supplementary Figs. 3–5). This consistency in spatial attention supported the clinical validity and robustness of the models. For the obstruction classification models (DISE-V-obs and DISE-OTE-obs), the airway lumen was highlighted because dynamic changes in airway morphology serve as the primary determinant of obstruction severity (Supplementary Figs. 3 and 4). Regarding the OTE cause classification model (DISE-OTE-cause), Grad-CAM demonstrated the model’s ability to spatially differentiate between distinct etiologies. In oropharyngeal lateral wall collapse, activation is concentrated on the lateral pharyngeal walls, including the tonsils. Tongue base collapse cases were marked by focused attention on the posterior tongue and vallecula. In contrast, epiglottic collapse predictions were concentrated at the epiglottic tip and base (Supplementary Fig. 5). Importantly, the model maintained minimal and focused activation in nonobstructive frames, further supporting its discriminative capability.

## Discussion

In this study, we developed and validated—both internally and externally—a deep learning algorithm to predict the degree and cause of upper airway obstruction using DISE videos. Because DISE typically evaluates obstruction in two primary regions—the velum and the OTE region—we developed two models for each view: DISE-V-obs and DISE-OTE-obs, to score the degree of obstruction. For the OTE region, we additionally developed a model—DISE-OTE-cause—to identify the primary obstructive structure among the oropharynx lateral wall, tongue base, and epiglottis.

The DISE-V-obs model demonstrated strong performance in predicting velum obstruction and was particularly effective in distinguishing between ‘no obstruction’ and ‘complete obstruction’. However, ‘partial obstruction’ remained challenging due to its intermediate nature and subtle visual features. This intermediate characteristic may lead to inter-clinician variability in ground truth labeling, with partial obstruction classifications being the most susceptible to inconsistency. The differences in predictive accuracy and F1 score between the internal and external datasets for DISE-V-obs were 3.8% and 2.3%, respectively. These small differences suggest that the DISE-V-obs model is robust across institutions. Although the ROC curve for the internal dataset indicated that the ‘no obstruction’ category was classified with high AUROC and minimal misclassification, a closer inspection of the per-class F1 score and confusion matrix revealed a discrepancy. Specifically, the ‘no obstruction’ class exhibited a high AUROC (0.95) but a relatively lower F1 score (81.9%), primarily due to classification challenges at the decision threshold. Given the class imbalance with a relatively low proportion of ‘no obstruction’ cases in the dataset, even occasional misclassifications of other obstruction classes as ‘no obstruction’ lead to increased false positives and substantially reduced precision. A similar discrepancy was observed in the external dataset (AUROC: 0.91, F1: 77.1%), further highlighting the influence of threshold calibration on final classification outcomes. Additionally, the intermediate nature of ‘partial obstruction’ posed particular challenges for the model’s classification performance, as this transitional category exhibits more subtle visual features than the more definitive obstruction states. This contributed to overlap between ‘partial obstruction’ and ‘complete obstruction’ being observed, particularly in the external dataset, likely reflecting increased variability in image quality and annotation standards across institutions.

The performance of the DISE-OTE-obs model was lower than that of the DISE-V-obs model. The OTE region comprises several structures—namely, the oropharynx lateral wall, tongue base, and epiglottis—making it more complex to predict obstruction degree in OTE videos than in velum videos. For the DISE-OTE-obs model, the external dataset showed a larger drop in accuracy (7.7%) than in F1 score (1.8%). This discrepancy is primarily explained by differences in class distribution between the internal and external datasets, particularly the substantially lower proportion of “no obstruction” cases in the external cohort. Because accuracy is highly sensitive to shifts in the majority class, misclassifications around adjacent severity levels had a greater cumulative impact on accuracy. In contrast, the F1 score, which balances precision and recall and is less affected by class imbalance, remained relatively stable. Accordingly, the small F1 gap suggests preserved discriminative performance, whereas the larger accuracy difference reflects dataset composition rather than a meaningful reduction in model robustness. Similar to the velum region, though more pronounced, in the internal dataset, the ‘complete obstruction’ class showed a moderate AUROC (0.82) but a low F1 score (60.4%), indicating substantial classification errors at the decision threshold. This finding reflects the challenge of accurately classifying this obstruction level, likely due to overlap with adjacent severity categories and class imbalance effects.

The DISE-OTE-cause model demonstrated strong performance in predicting the primary cause of obstruction in the OTE region during internal validation. However, its performance decreased in the external validation, with a considerable performance gap between the internal and external datasets. This discrepancy may be attributed to variability in the ground truth labels. Ground truth values for the primary cause of OTE obstruction were determined by two sleep specialists: one from the medical center where the DISE was performed, and the other from a different center participating in the study. In contrast to obstruction degree—where well-defined criteria exist (i.e., 50% and 90%)—the determination of the primary cause of obstruction is relatively subjective, which may explain the substantial performance differences between internal and external validation for the DISE-OTE-cause model. Specifically, DISE-OTE-cause exhibited reduced performance in identifying epiglottic obstruction as the primary cause of OTE obstruction in the external dataset. Within the VOTE classification system, isolated epiglottic obstruction is the least common subtype, with reported frequencies ranging from 3.5% to 14.4%^18–21^. Intermittent epiglottic folding observed during DISE may be classified as isolated epiglottic collapse by some sleep specialists, while others may not interpret it as such—depending on their individual definitions of this condition. There is currently no consensus on the threshold frequency or presentation of epiglottic folding during DISE that should qualify as isolated epiglottic collapse. Furthermore, less experienced specialists may misinterpret secondary obstruction caused by the tongue base or oropharynx lateral wall as primary epiglottic collapse^22^. In contrast, experienced sleep specialists may instinctively identify the primary site of obstruction, whereas deep learning systems—operating through unknown detection mechanisms—may adopt alternative strategies to localize the primary cause of obstruction in the OTE region.

The OTE region includes multiple anatomical structures, such as the oropharynx lateral wall, tongue base, and epiglottis, and independently evaluating the obstruction of these structures may not be optimal in a deep learning model. For instance, an airway obstruction involving the epiglottis is not necessarily caused by the epiglottis itself; rather, posterior displacement of the tongue base may lead to epiglottic involvement, which could be overlooked by an independent evaluation approach. To address this issue, we accounted for the interrelationships among these anatomical structures during training of the DISE-OTE-obs and DISE-OTE-cause models. Instead of assessing obstruction severity for each structure based on the VOTE classification, we defined the obstruction degree within the OTE region in terms of overall airway compromise and developed a model to identify the primary cause of airway obstruction. Since identifying the primary cause is critical for determining a patient’s treatment plan in clinical practice, this approach is expected to have strong potential for direct clinical application. To ensure optimal evaluation that considers both anatomical characteristics and the clinical assessment objective—whether obstruction degree or cause—we developed three models: DISE-V-obs, DISE-OTE-obs, and DISE-OTE-cause. The F1 scores for these models were 84.7%, 74.7%, and 88.2%, respectively, all of which exceed the performance reported in a previous study^22^. To minimize selection bias and enhance the representativeness of our dataset, we intentionally included all eligible video clips that met the minimum evaluation criteria, even if only one of the velum or OTE segments was available for a given patient, instead of limiting the dataset to patients with completely paired recordings. As a result, some patients in our cohort contributed only one velum or OTE video clip for analysis. To ensure that this inclusive approach did not skew the results, we conducted a sensitivity analysis restricted to the subset of patients for whom both velum and OTE clips were available (2845 DISE videos from 1540 patients; 2263 internal and 582 external clips). The performances of the DISE-V-obs, DISE-OTE-obs, and DISE-OTE-cause models in this subset were comparable to those of the main analysis, further supporting the robustness and generalizability of our findings (Supplementary Fig. 6).

Although the architecture employed in this study, EfficientNet-B2 combined with Attention-based multiple instance learning (MIL), is not novel in the field of machine learning, we deliberately selected this model as the most suitable for our clinical objective. Rather than proposing a new neural architecture, we focused on applying a well-established and high-performance model to a clinically complex task using a large, heterogeneous, and multicenter dataset. To further substantiate the methodological validity of this choice, we conducted baseline comparisons evaluating alternative multi-frame aggregation strategies. Specifically, we compared our attention-based aggregation mechanism with two commonly used MIL baselines—mean pooling and max (voting) pooling. As shown in Supplementary Fig. 7, the attention-based MIL model—by adaptively assigning higher weights to informative frames while suppressing irrelevant ones—consistently outperformed both baselines across accuracy and F1 score in internal and external validation, demonstrating improved robustness against noisy frames and stronger generalizability. Furthermore, an ablation analysis comparing the Attention MIL model with and without class-weighted cross-entropy loss demonstrated that explicitly addressing class imbalance consistently resulted in higher accuracy and F1-scores across all three models (DISE-V-obs, DISE-OTE-obs, and DISE-OTE-cause; Supplementary Fig. 8). This finding further reinforces the robustness of our proposed approach, particularly in clinically realistic settings where class distributions are inherently imbalanced. Building upon these performance improvements, we additionally aimed to enhance model transparency and interpretability by leveraging Grad-CAM visualizations, which provided intuitive insights into the spatial focus of the network predictions. The alignment between the model’s spatial attention and clinically relevant anatomical landmarks provides a qualitative confirmation of its decision-making process and confirms that the network focuses on clinically relevant features. These visual explanations significantly enhance prediction transparency and can strengthen clinician trust in the system.

To our knowledge, this is the largest study on an automatic scoring system for DISE and the only study to date that has been both internally and externally validated. High inter-rater variability in DISE scoring remains a persistent challenge, undermining its clinical utility and often leading to inappropriate treatment selection. Previous studies have reported inter-rater agreement with kappa values ranging from 0.40 to 0.60 for the velum and from 0.34 to 0.52 for the OTE region^11,13–15^. In 2023, Hanif et al. developed an automatic scoring model using deep learning based on 281 DISE video clips, achieving kappa values of 0.55 for the velum and 0.43 for the OTE region; however, external validation was not performed^22^. In the present study, the kappa values were substantially higher 0.78 for the velum and 0.66 for the OTE region and, notably, external validation was also conducted. Supplementary Table 3 provides a comparative summary of our model’s performance alongside prior studies that have either developed prediction models or reported inter-rater agreement for DISE scoring. The clinical workflow integration of our deep learning-based DISE scoring system offers a practical solution to address inter-rater variability. Rather than replacing physician judgment, the system is designed to function as a clinical decision support tool, providing standardized and reproducible scoring that can enhance interpretation consistency, particularly for less experienced clinicians. Integration may occur either in real-time during DISE procedures—offering automated scoring alongside live endoscopy—or during post-procedural review to support treatment planning. By enhancing the accuracy of obstruction assessment, the system has the potential to guide patient-specific management strategies more effectively. Importantly, successful deployment will require prospective validation to assess its impact on clinical decision-making and outcomes, while ensuring that final diagnostic authority remains with the clinician.

This study has some limitations. First, the characteristics of the video clips were heterogeneous. Although multicenter data collection is a major strength of this study, DISE was performed by different rhinologists across centers, and variations in DISE technique, clip quality, and frame alignment may have occurred. To address this, we standardized all video clips to a duration of 5 seconds at five frames per second (totaling 25 frames), applied pixel-value normalization and inversion, and extracted motion-based regions of interest to enhance consistency across the dataset. Second, we acknowledge that our approach involved manual selection of representative video segments corresponding to the velum and OTE phases. While we agree that a fully automated analysis of the entire DISE video is the ultimate goal, we believe that developing robust models for each phase using representative, high-quality clips is a necessary and practical first step. Finally, inter-rater variability may have influenced the ground truth labels. Although scoring was provided by up to three sleep specialists, inter-rater variability was not fully controlled, which was particularly evident in the DISE-OTE-cause model.

In conclusion, this study demonstrates the feasibility and potential of using deep learning to analyze the degree of upper airway obstruction in sleep apnea and predict its primary causes. By providing clinicians with an automatic DISE scoring system as supporting information, we anticipate improved diagnostic consistency and reduced inter-rater variability, ultimately enabling more accurate treatment planning for patients with OSA.

## Methods

### Data acquisition

**Fig. 6:**
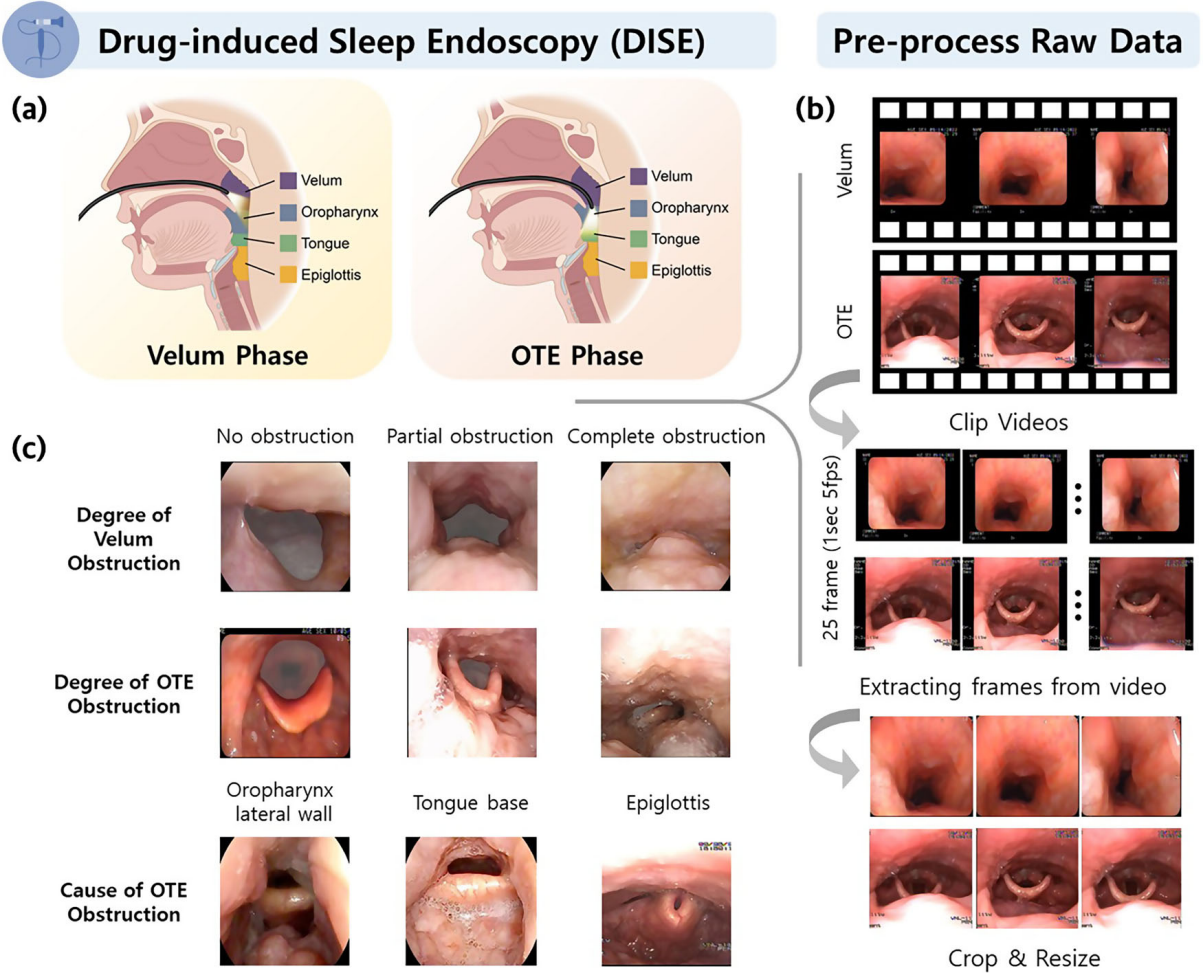
Overview of DISE data structure and preprocessing workflow. **a** DISE is performed in two phases—Velum phase and OTE (oropharynx, tongue base, epiglottis) phase—according to the anatomical position of the inserted endoscope. **b** Examples of anatomical classifications for the obstruction degree at the velum and OTE regions (no obstruction, partial obstruction, complete obstruction), with airway lumen boundaries marked, as well as the cause of obstruction within the OTE region (oropharynx lateral wall, tongue base, epiglottis). **c** Preprocessing pipeline including video clipping, frame extraction, and resizing. Each clip was standardized to 5 seconds with five frames per second (25 frames total). Motion-based regions of interest in individual frames were extracted, followed by normalization, inversion, cropping, and resizing to 256 × 256 pixels. This ensured uniform input structure and enhanced clinical relevance.

DISE videos were obtained from 1904 patients across five representative regional hospitals in South Korea: Seoul National University Hospital, Pusan National University Hospital, Pusan National University Yangsan Hospital, Chonnam National University Hospital, and Kyungpook National University Chilgok Hospital. The inclusion criteria for DISE examinations were patients suspected of having a sleep-related breathing disorder, characterized by symptoms such as daytime sleepiness, witnessed sleep apnea, or fatigue. All severity levels of OSA were included, ranging from normal (AHI < 5) to severe. Pediatric cases were excluded. DISE was performed by sleep specialists at each hospital in accordance with the procedural guidelines provided by the European position paper^23,24^. The procedure was conducted either in the operating room (Pusan National University Hospital, Chonnam National University Hospital, and Kyungpook National University Chilgok Hospital) or in the endoscopy room (Seoul National University Hospital and Pusan National University Yangsan Hospital). Following upper airway evaluation while the patient was awake, sedation was induced using target-controlled infusion of either propofol (starting dose: 2.0–2.5 μg/mL) or midazolam (0.05 mg/kg). Patients were then evaluated in both the supine and lateral positions during induced sleep. All videos were recorded using a flexible fiberoptic endoscope [Olympus OTV-S200 (Olympus Corporation, Tokyo, Japan) or Pentax VNL-1190STK (Pentax Medical, Tokyo, Japan)] and a video processor [Pentax EPK-3000 or EPK-5000 (Pentax Medical, Tokyo, Japan)]. Selected video clips captured the velum and OTE (oropharynx, tonsils, and epiglottis) phase, including both baseline and obstructed airway states (Fig. 6a).

Across the five participating institutions, 4437 Velum and 4190 OTE video clips were initially collected. The number of OTE clips was ~5.6% lower than that of the Velum clips, primarily due to the anatomical proximity between the velum and OTE regions in certain patients. In these cases, the absence of significant velum obstruction allowed adequate OTE visualization during velum examination, reducing the need for separate OTE recordings. Of these, 3744 Velum clips (84.4%) and 3098 OTE clips (73.9%) were included in the final analysis. The remaining 693 Velum clips (15.6%) and 1092 OTE clips (26.1%) were excluded because of the absence of polysomnogram data, excessive salivation obscuring visualization, insufficient brightness for structural identification, low resolution, or excessive motion due to rapid endoscope manipulation. OTE clips were more frequently excluded because of severe artifacts (e.g., gag reflex, excessive salivation, or incomplete visualization of key structures such as the tongue base). However, to reflect real-world clinical conditions, we retained clips with mild-to-moderate artifacts such as slight saliva interference or suboptimal lighting, which are commonly encountered during DISE. Although DISE is typically performed in the supine position, some institutions have also acquired segments in the lateral or re-supine position. As a result, a single patient could contribute more than one video clip per anatomical region (velum or OTE), depending on the position. Consequently, the total number of video clips exceeded that of the individual patients. In total, the clips used in this study were derived from 1904 patients, of whom 1540 contributed both Velum and OTE clips, 234 contributed only Velum clips, and 130 contributed only OTE clips.

The ground truth labels for obstruction degree and cause were determined by two sleep specialists from different hospitals based on the VOTE classification system^25^. In cases of disagreement, a third sleep specialist provided the final decision. All labels were assigned through manual visual assessment, with the ground truth based entirely on expert consensus, and not on automated estimations. Obstruction degree was graded on a three-point scale: 0 (no obstruction), 1 (partial obstruction), and 2 (complete obstruction or collapse). “No obstruction” was defined as <50% airway narrowing, “partial obstruction” as 50–90% narrowing, and “complete obstruction” as >90% narrowing of the upper airway cross-sectional area (Fig. 6b)^26^. In contrast to the velum video clips, in which the primary obstruction site is consistently the velum, the OTE video clips may exhibit primary obstruction at the oropharyngx lateral wall, tongue base, or epiglottis (Fig. 6b). In the original VOTE classification, the degree of obstruction is assessed for each site individually, and the primary cause is typically inferred based on the overall pattern. Deep learning has made it feasible to design a classification scheme that supports a more direct estimation of the dominant anatomical cause. Accordingly, we established ground truth labels for velum obstruction, OTE obstruction, and the primary cause of OTE obstruction—forming a structured adaptation of the VOTE classification. This refinement reflects the standard procedural approach for DISE, which is typically conducted in two sequential phases: velum and OTE. While preserving the original anatomical definitions and obstruction criteria of VOTE, this framework facilitates a clinically intuitive representation of the two key questions addressed by DISE: the degree of airway collapse and its primary anatomical cause. Consensus on obstruction degree and OTE cause was reached through more than ten online and offline meetings among participating sleep specialists to establish detailed specifications within the VOTE classification framework, such as anatomical boundaries, clinical criteria for determining the primary cause of obstruction, and specific threshold values for severity classification. This study was conducted in accordance with the Declaration of Helsinki and relevant ethical guidelines and regulations. Ethical approval for this retrospective study was obtained from the institutional review boards of all participating hospitals, including the Seoul National University Hospital (IRB No. 2305-015-1428), Kyungpook National University Hospital (IRB No. 2023-08-015), Pusan National University Hospital (IRB No. 2305-011-127), Pusan National University Yangsan Hospital (IRB No. 05-2023-141), and Chonnam National University Hospital (IRB No. 2023-182). The requirement for informed consent was waived by all institutional review boards due to the retrospective nature of the study.

### Pre-processing

To predict the degree of obstruction in the velum and OTE regions, we designed an efficient preprocessing pipeline that considered both computational resource constraints and the nature of the labeling data. Each full video clip contained only a single label, which would have resulted in high computational cost if used in its entirety for deep learning model training. To address this issue, we adopted a clip-based approach where representative video segments (mean duration: 17.3 ± 3.4 seconds) were selected for each region (velum or OTE), and frames were extracted at a rate of five frames per second (resulting in an average of 86 frames per clip). All video clips consisted of three-color channels (R, G, and B), and the external view captured before endoscope insertion was removed. To enhance clinical relevance and reduce noise, we applied motion-based frame selection by computing inter-frame pixel differences to generate motion maps. Periods with low cumulative motion—such as static endoscopic views, saliva pooling, or minimal airway movement—were discarded based on a predefined threshold. Using the generated motion maps, attention-based weighting was then applied to select 25 high-quality frames per clip, enabling the model to focus on dynamic airway features while standardizing all inputs to 5-second segments per clip (Fig. 6c). Regions exceeding a predefined pixel-value threshold—based on the pixel distribution—were retained for further processing. This process preserves frames that contain meaningful motion while removing irrelevant or static background areas, which improves both the clarity of the input data and the efficiency of model training.

For each selected frame, motion-rich regions were localized using their boundary coordinates, and these ROIs were then cropped and resized to 256 × 256 pixels while maintaining the original aspect ratio as much as possible to preserve anatomical structure. The pre-processed frames were converted into tensors, with all pixel values normalized to the range [0, 1]. Pixel values were then inverted (1−value) to enhance contrast and facilitate the model’s recognition of key features, particularly areas of airway closure. In combination, the normalization and inversion steps reduced data noise, facilitated stable model training, and mitigated overfitting risks. This preprocessing procedure was applied to both the internal and external datasets to ensure consistency across institutions.

The degree of obstruction of the velum and OTE regions was categorized into three classes based on the degree of airway narrowing: no obstruction, partial obstruction, and complete obstruction. Of the total dataset, 2422 velum region samples were used for model training, and the remaining 605 samples were used as evaluation data to verify performance. For the OTE region, 1882 samples were used for training, and 470 samples were used for validation. The causes of obstruction in the OTE region were categorized into three classes based on the anatomical origin of airway narrowing: oropharynx lateral walls, tongue base, and epiglottis. This categorization applied only to cases exhibiting partial or complete obstruction. A total of 918 samples were used to train the model, and 229 samples were used for performance validation.

### Deep learning architecture configuration

**Fig. 7:**
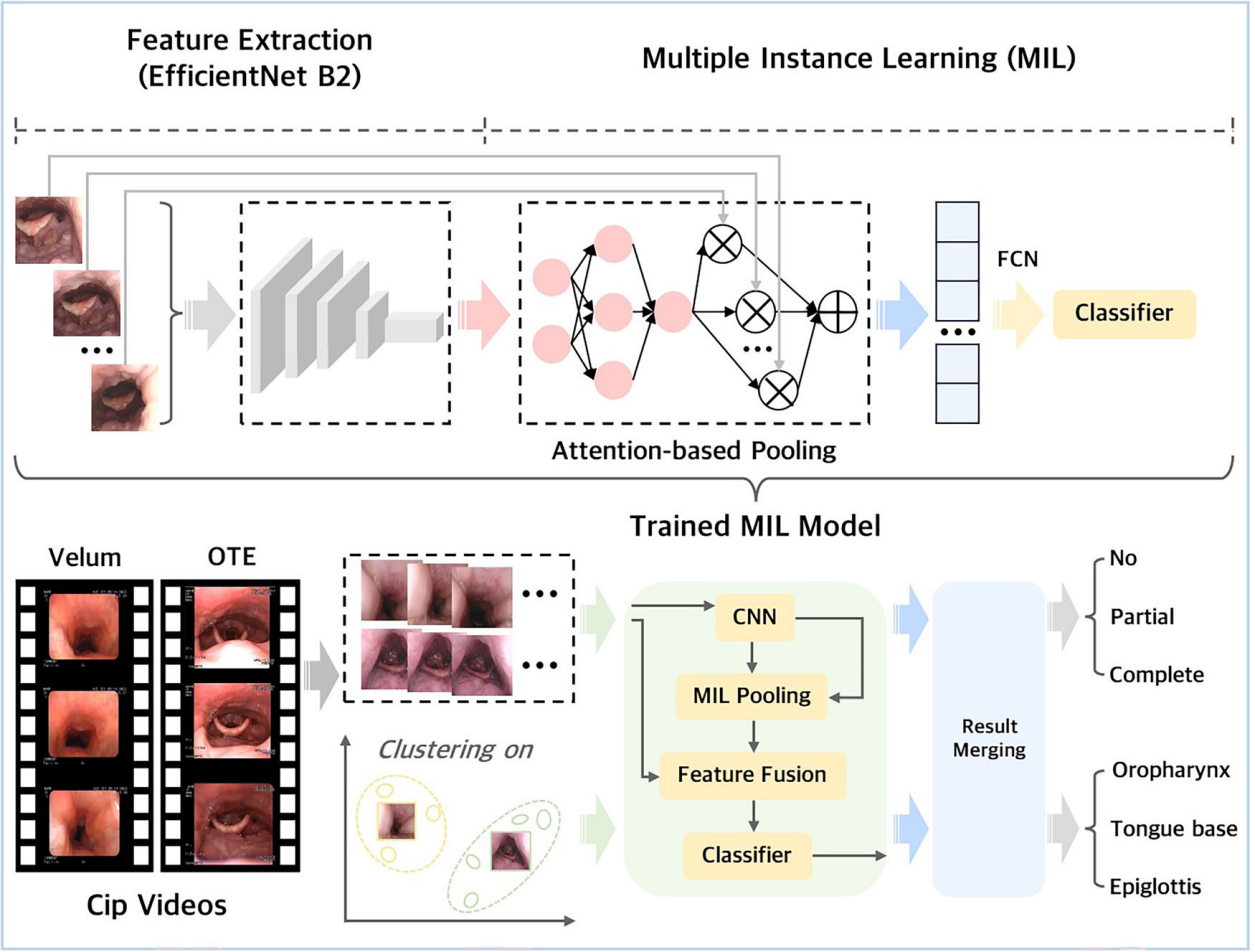
Architecture of the proposed deep learning model for obstruction classification. The model consists of an EfficientNet-B2 backbone integrated with an attention-based multiple instance learning (MIL) framework. The design enables frame-wise feature extraction and video-level aggregation for obstruction degree and cause prediction. The attention mechanism assigns importance weights to each frame, allowing the model to focus on diagnostically relevant regions while suppressing irrelevant artifacts. Final predictions are made through a combination of fully connected (FCN) and convolutional (CNN) modules tailored to both obstruction severity and cause classification tasks.

In this study, we developed a deep learning model based on the MIL framework to predict the degree of obstruction—categorized as no, partial, or complete—in the velum and OTE regions, as well as the primary cause of obstruction in the OTE region (Fig. 7). Specifically, we developed deep learning models—DISE-V-obs and DISE-OTE-obs—to predict the degree of obstruction in the velum and OTE regions, respectively. In addition, we constructed a model—DISE-OTE-cause—to predict the cause of obstruction in the OTE region, with a focus on three anatomical structures: the oropharynx lateral wall, tongue base, and epiglottis. This architecture employs EfficientNet-B2—a lightweight yet high-performance feature extractor—as the backbone for extracting high-level features from input images. We modified EfficientNet-B2 by removing its final fully connected layer so that it could output feature vectors instead of classification scores. An attention mechanism was then incorporated to select a fixed number of diagnostically meaningful frames from the extracted frames, thereby standardizing the input length across all clips. The attention mechanism first computed the importance weights for each frame and then normalized these weights using the SoftMax function. The normalized weights were subsequently applied to the frame-level features to compute a single aggregated feature vector. Through this process, the model automatically learned to focus on frames containing critical pathological cues, such as pharyngeal wall narrowing, posterior displacement of the tongue base, or epiglottic collapse, while downweighting frames with minimal airway changes or irrelevant artifacts, such as motion blur, saliva, or poor visibility. In addition to evaluating frames individually, the model captured temporal dynamics across clips, allowing it to infer changes in the airway structure over time, such as gradual collapse or reopening patterns, which are crucial for differentiating obstruction severity. This attention-based filtering allowed the model to concentrate on the most diagnostically meaningful frames without requiring explicit segmentation or manual frame selection. The model was trained using class-weighted cross-entropy loss to address class imbalance with dropout and ReLU activation for regularization. We also applied the sharpness-aware minimization (SAM) algorithm to promote stable and generalizable learning by avoiding sharp minima, which could lead to overfitting.

Grad-CAM is a widely used visualization technique that generates heatmaps indicating the most influential regions for a model’s prediction, by back-propagating gradients through convolutional layers^27^. We visualized the attention-guided focus of the model using Grad-CAM, which demonstrated that the model consistently highlighted anatomically relevant obstructive regions—including the collapsible airway and surrounding soft tissues—aligned with clinical expectations. These findings corroborated the model’s attention-based selection mechanism and provided visual validation that its focus corresponded to clinically meaningful features at the frame level.

### Experiment setup

In this study, we established a computational environment to predict the degree and primary cause of obstruction in the velum and OTE regions. All experiments were conducted on an Ubuntu 20.04.6 LTS operating system (Canonical, London, United Kingdom), using Python (version 3.9.0) and PyTorch (version 2.4.0) as the primary development frameworks for data processing and model implementation. The hardware environment consisted of a high-performance system equipped with four NVIDIA A100 (40 GB) graphics processing units (NVIDIA Corporation, Santa Clara, CA), an AMD EPYC 7513 32-core 2.5 GHz processor, and 512 GB of RAM. Model training for predicting the degree and primary cause of obstruction in the velum and OTE regions was conducted for up to 300 epochs with a batch size of four and a learning rate of 2e − 5. For network optimization, we used cross-entropy loss and the Adam optimizer. Additionally, an early stopping strategy was implemented to prevent overfitting during training. Supplementary Table 4 provides a summary of the key hyperparameters and reproducibility information.

### Performance evaluation

The performance of the prediction models for the degree of velum and OTE obstruction, as well as the cause of OTE obstruction, was evaluated using fivefold cross-validation. To ensure the validity of the model assessment and prevent data leakage, cross-validation was strictly conducted on a clip-wise basis. All DISE video clips were assigned exclusively to either the training or the test set within each fold. This ensured that no clips appeared in either set simultaneously, thereby minimizing the risk of overfitting to clip-specific visual features and supporting generalizability to unseen data. To further evaluate the model’s robustness across institutions, we performed external validation using an entirely independent dataset collected from separate institutions. This external dataset was not involved in any stage of model training, hyperparameter tuning, or cross-validation, and was used solely for evaluating the final model performance. Specifically, the external dataset was applied independently to the model trained in each fold of internal cross-validation. Performance metrics from these five evaluations were aggregated to report the average performance along with 95% confidence intervals, thereby providing a more robust estimate of model generalizability to unseen institutional data. Evaluation metrics included accuracy, sensitivity, specificity, F1 score, Cohen’s kappa, and AUROC. Except for AUROC, all metrics were calculated using micro-averaging, which aggregates the contributions of all classes. In multiclass evaluation, the traditional definitions of positive and negative classes are not directly applicable to our three-class classification tasks. In this setting, true positives (TP), false positives (FP), true negatives (TN), and false negatives (FN) were computed for each class using a one-vs-rest approach. These counts were then summed across all classes and used to calculate sensitivity (TP/[TP + FN]), specificity (TN/[TN + FP]), accuracy (correct predictions/total predictions), and F1 score (harmonic mean of precision and recall). Cohen’s kappa was also calculated based on the aggregated agreement values across all classes. Micro-averaging allows all predictions to contribute equally, mitigating class imbalance and yielding a comprehensive assessment of the overall model performance. Class-wise performance metrics, including accuracy, sensitivity, specificity, and F1 score, are included separately for clearer interpretability.

## Supplementary information

41746_2026_2673_MOESM1_ESM

## Data Availability

The publicly available DISE Video Dataset can be accessed through AIHub, a Korean national AI data portal, at the following URL: https://www.aihub.or.kr/aihubdata/data/view.do?dataSetSn=71689 (accessed on 11 June 2025). To access the data, users must first create a free account and agree to the terms of use. Access is granted upon request through the AIHub system. This dataset includes annotated DISE video clips intended for the development and validation of AI-based models for upper airway obstruction analysis.

## Code availability

The full source code for the DISE-based obstruction prediction models, including the preprocessing scripts, training pipelines, and evaluation tools, is publicly available at https://github.com/Ysbaik/AttentionMIL.git. The experiments were conducted using Python 3.9.0 and the following open-source libraries: torch = 2.4.0, opencv-python = 4.5.5.64, matplotlib = 3.3.4, tqdm = 4.59.0, pandas = 1.3.5, numpy = 1.21.5.
