# Supplementary material for: Deep learning-based automatic scoring of drug-induced sleep endoscopy in obstructive sleep apnea
Source: NPJ Digit Med. 2026 Apr 28;9:504. doi: 10.1038/s41746-026-02673-8 (PMC13332034; doi:10.1038/s41746-026-02673-8)
Supplement: Supplementary file 1 — 41746_2026_2673_MOESM1_ESM [file 41746_2026_2673_MOESM1_ESM.pdf]

Supplementary Information

Deep learning-based automatic scoring of drug-induced sleep endoscopy in obstructive sleep apnea

Jin Youp Kim[1†], Sue Jean Mun[2†], Young Seo Baik[3†], Young Seop Lee[4], Young Jae Kim[5], Jayoung Oh[6], Gwanghui Ryu[7], Chung-Man Sung[8], Sung Jae Heo[9], Hyung Chae Yang[8], Hyun Jik Kim[10], Hyo Yeol Kim[7], Kyu-Sup Cho[11], Kwang Gi Kim[12*], Chae-Seo Rhee[10*]

***Corresponding authors:** Chae-Seo Rhee (csrhee@snu.ac.kr), Kwang Gi Kim (kimkg@gachon.ac.kr)

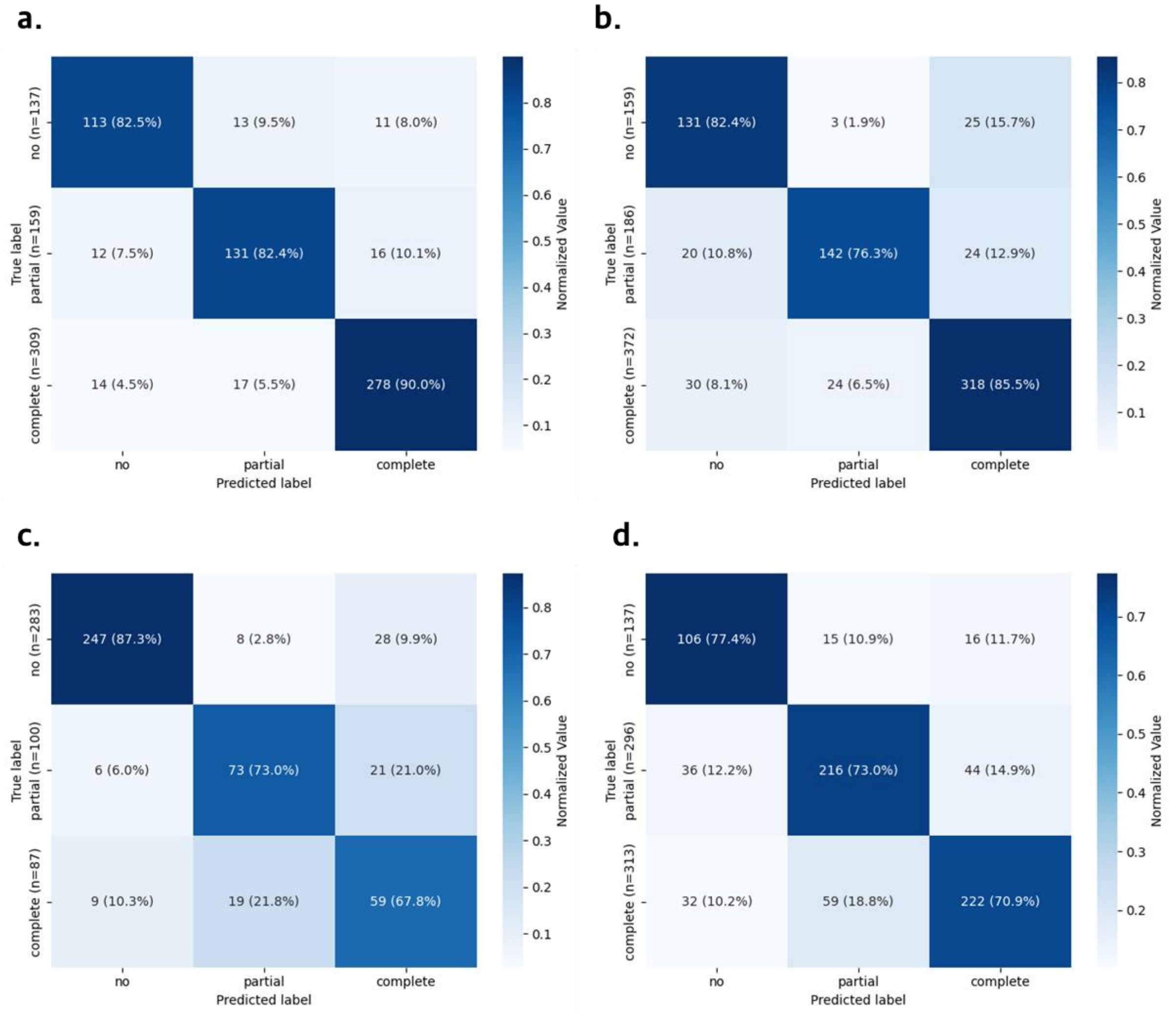

Supplementary Figure 1. Confusion matrices for obstruction degree classification in the Velum and OTE region. Confusion matrices were row-normalized to visualize the classification distribution per true class. The number of samples (n) in each true class is also displayed alongside each row label for clarity. (a) Velum internal dataset, (b) Velum external dataset, (c) OTE internal dataset, (d) OTE external dataset.

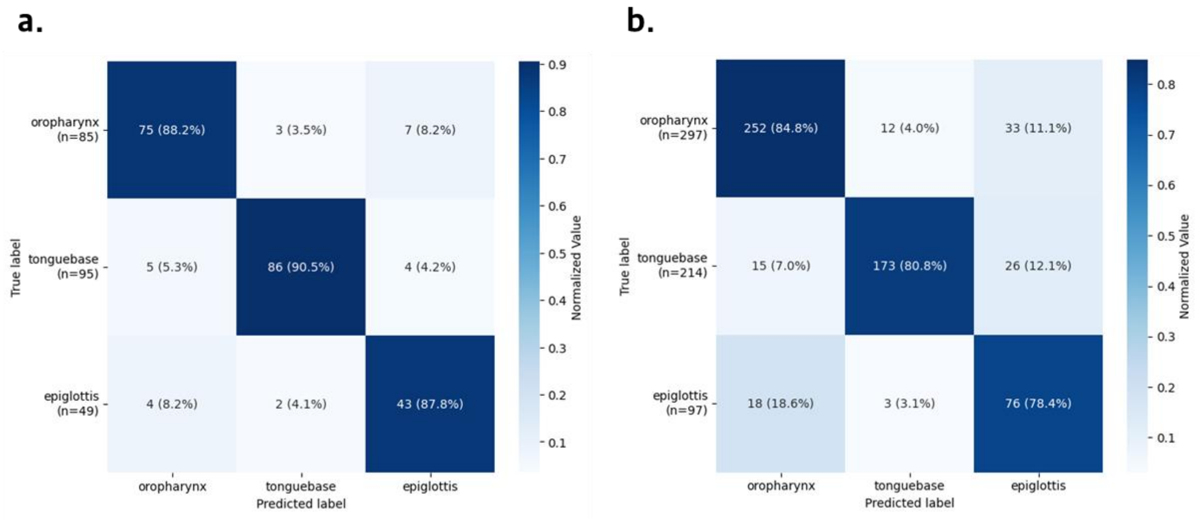

Supplementary Figure 2. Confusion matrices for obstruction cause classification in the OTE region. Confusion matrices are row-normalized to visualize the classification distribution per true class. The number of samples (n) in each true class is also displayed alongside each row label for clarity. (a) Internal dataset, (b) External dataset.

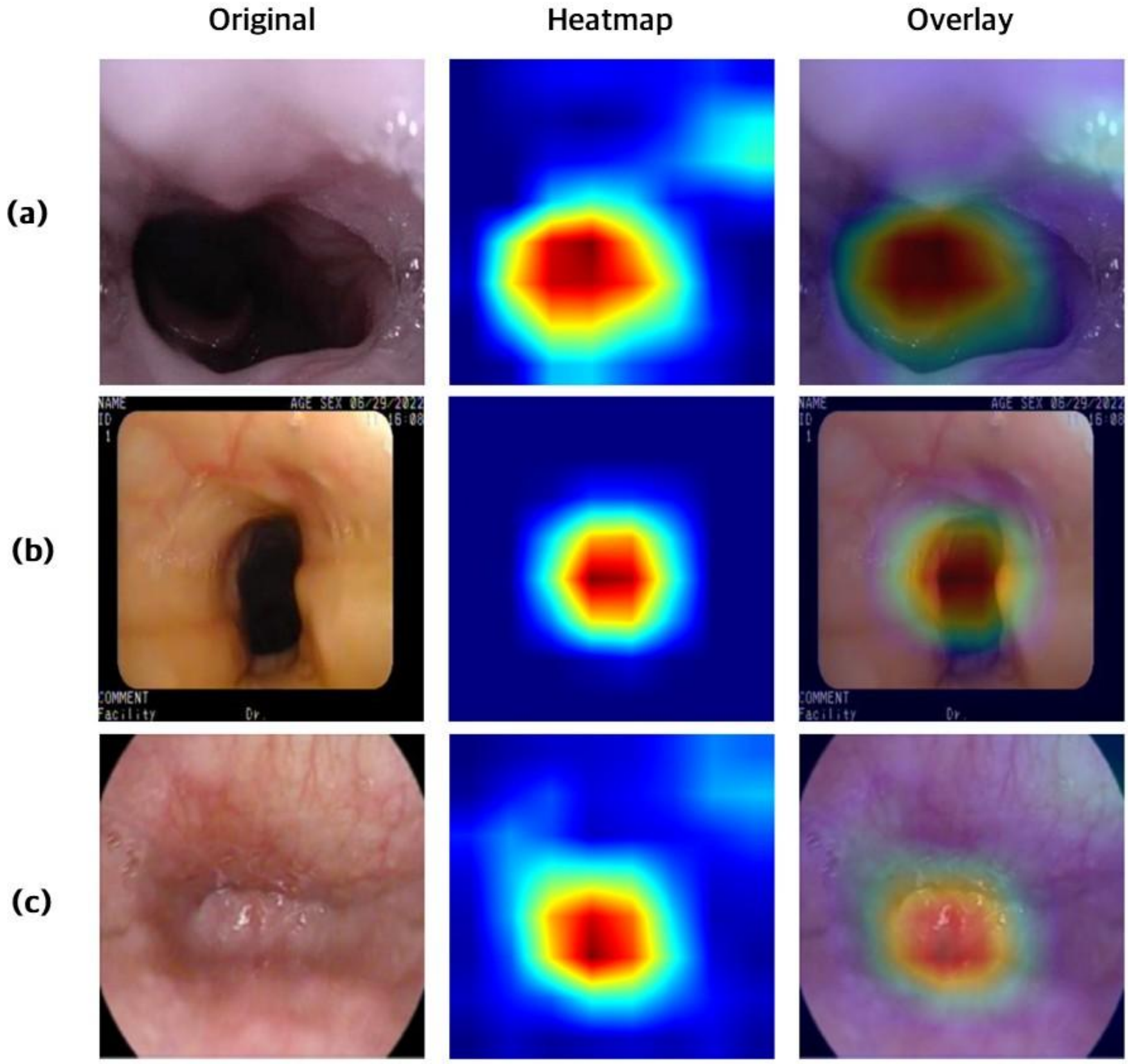

Supplementary Figure 3. Grad-CAM visualizations of the DISE-V-obs model for obstruction degree classification in the velum region. (a) no obstruction, (b) partial obstruction, and (c) complete obstruction. For each case, the original image, Grad-CAM heatmap, and overlay are shown from left to right. The model consistently highlights the airway lumen and adjacent soft tissues.

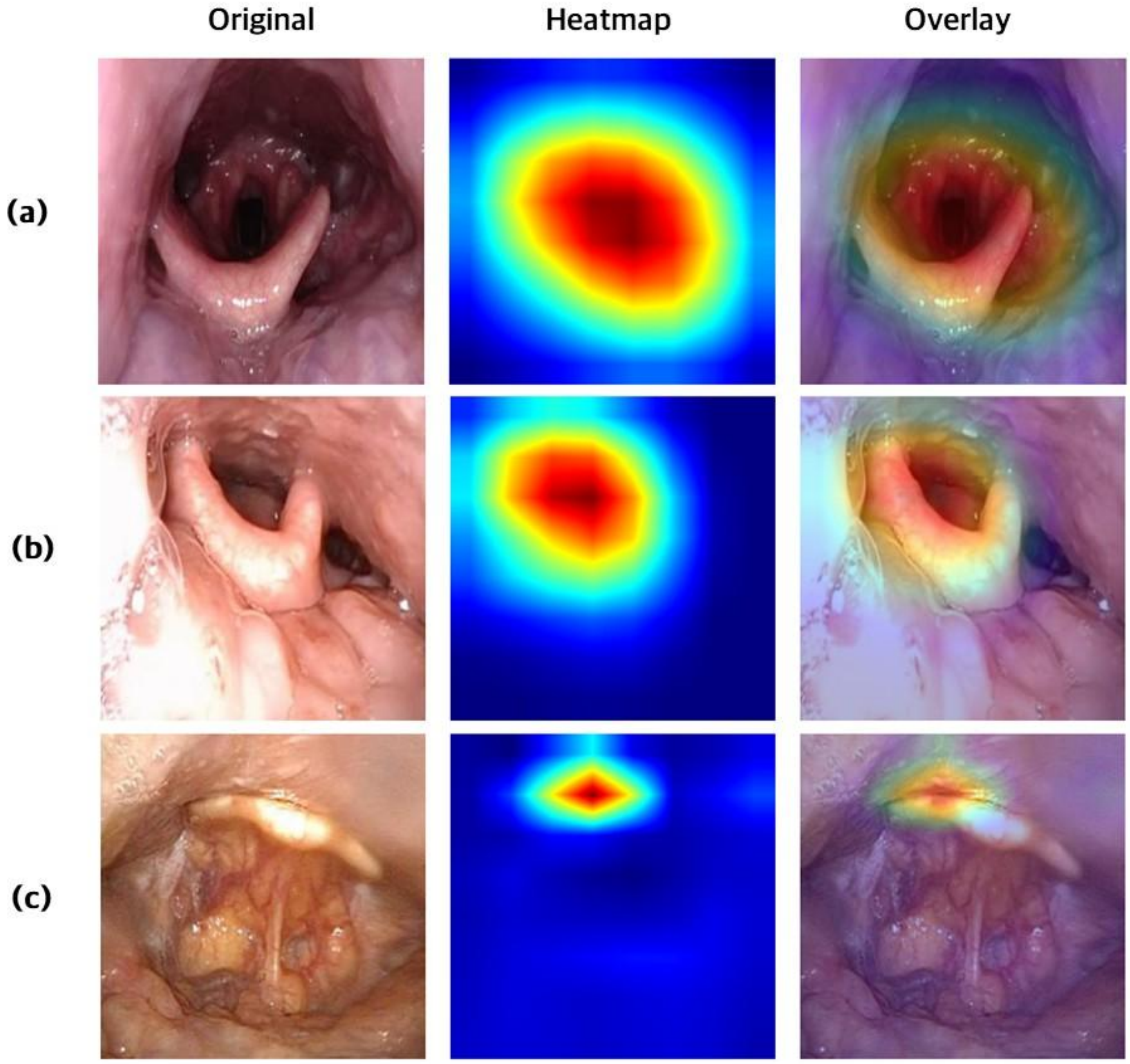

Supplementary Figure 4. Grad-CAM visualizations of the DISE-OTE-obs model for obstruction degree classification in the OTE region. (a) no obstruction, (b) partial obstruction, and (c) complete obstruction. From left to right, each panel displays the original endoscopic frame, Grad-CAM heatmap, and the overlayed image. The model consistently emphasizes the airway lumen and adjacent collapsing structures.

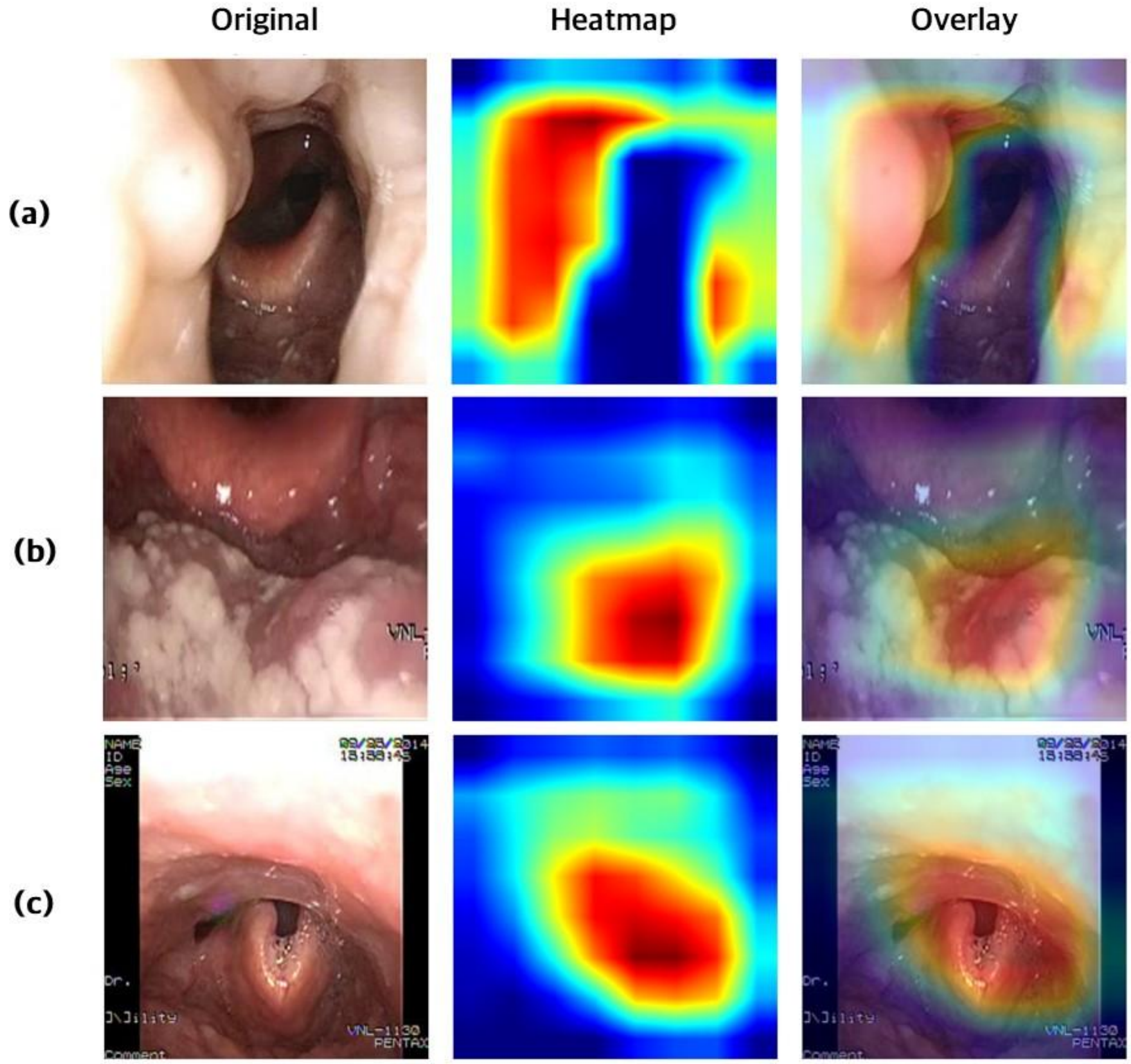

Supplementary Figure 5. Grad-CAM visualizations of the DISE-OTE-cause model for classifying primary causes of obstruction in the OTE region. (a) oropharynx lateral wall obstruction, (b) tongue base obstruction, and (c) epiglottic obstruction. From left to right, each panel shows the original endoscopic image, the corresponding Grad-CAM heatmap, and the overlayed visualization. The model successfully localized spatially relevant features: activations in (a) were concentrated on the oropharynx lateral walls, (b) emphasized the posterior tongue and vallecula, and (c) focused on the epiglottic tip and base. These findings support the model's interpretability and its ability to distinguish among obstruction causes based on anatomical localization.

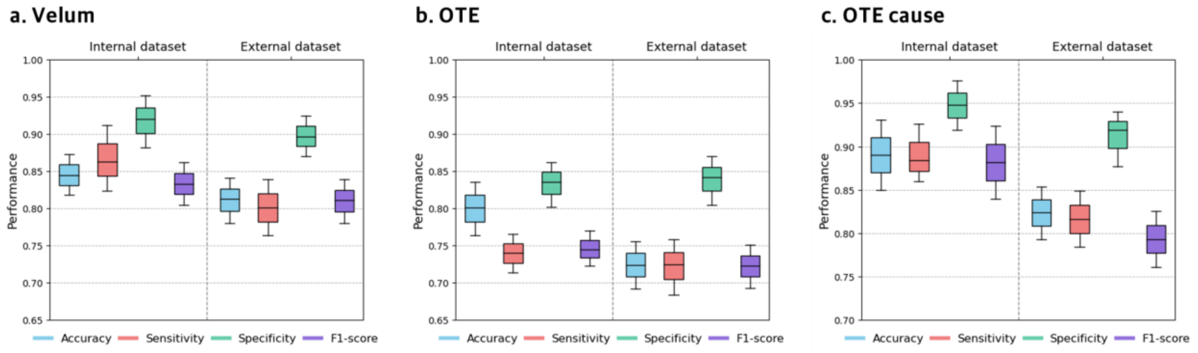

Supplementary Figure 6. Sensitivity analysis based on a subset of 2,845 DISE video clips from 1,540 patients, each providing both Velum and OTE segments. The subset includes 2,263 clips from the internal dataset and 582 from the external dataset. The performance metrics of the three models—DISE-V-obs, DISE-OTE-obs, and DISE-OTE-cause—were evaluated using this subset to assess the robustness and generalizability of the main findings. The results were comparable to those from the main analysis, supporting the validity of including unpaired clips in the primary dataset.

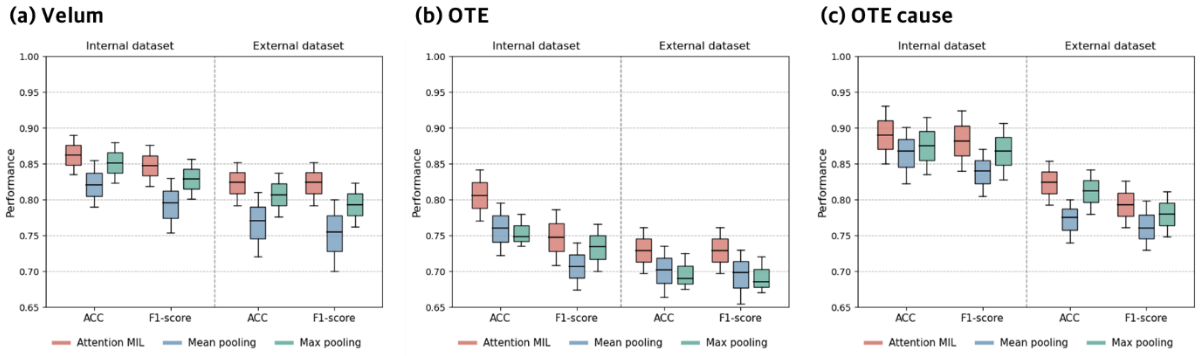

Supplementary Figure 7. Performance comparison between the proposed Attention-based MIL model and baseline MIL aggregation strategies (Mean pooling and Max pooling) for predicting velum and OTE obstruction outcomes on both internal and external datasets. (a) DISE-V-obs (b) DISE-OTE-obs (c) DISE-OTE-cause. Mean pooling uniformly averages features across all frames and therefore tends to dilute clinically meaningful frame-level signals, while Max pooling relies only on a single highest-scoring frame and is more susceptible to noise. In contrast, the Attention-based MIL model adaptively assigns weights to informative frames and suppresses irrelevant ones, enabling more reliable feature integration. The Attention MIL model demonstrated consistently superior performance with higher accuracy and F1-score and reduced variance compared with the baseline methods.

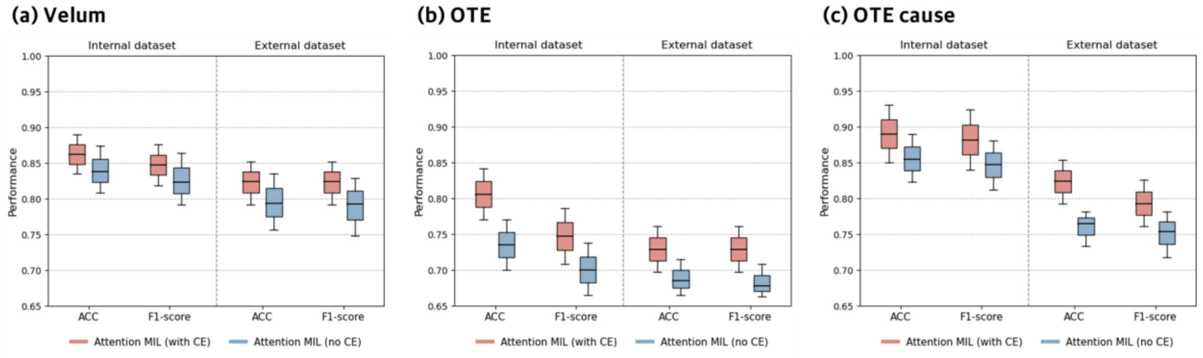

Supplementary Figure 8. Ablation analysis of class-imbalance handling in the Attention-based MIL model. (a) DISE-V-obs (b) DISE-OTE-obs (c) DISE-OTE-cause. For each task (DISE-V-obs, DISE-OTE-obs, and DISE-OTE-cause), boxplots show a comparison of model performance with class-weighted cross-entropy loss versus the same architecture without class weighting across internal and external datasets. The class-weighted variant, which assigns higher weights to under-represented classes based on their inverse frequency in the training set, consistently achieved higher accuracy and F1-scores.

Supplementary Table 1. Per-class performance metrics of DISE-based models for obstruction degree prediction in the velum and OTE regions. This table presents the class-wise accuracy, sensitivity, specificity, and F1-score of the obstruction degree classification models. Metrics were reported separately for the three obstruction categories (no, partial, and complete) in both the velum and OTE regions and evaluated using internal and external datasets.

Performance Metrics		**Velum Obstruction Degree**		**OTE Obstruction Degree**	
		**Internal Dataset**	**External Dataset**	**Internal Dataset**	**External Dataset**
**Accuracy**	**no obstruction**	0.825 (0.753–0.879)	0.824 (0.757–0.875)	0.873 (0.834–0.910)	0.774 (0.697–0.836)
	**partial obstruction**	0.824 (0.757–0.875)	0.763 (0.697–0.819)	0.731 (0.644–0.817)	0.730 (0.676–0.777)
	**complete obstruction**	0.900 (0.861–0.928)	0.855 (0.815–0.887)	0.678 (0.573–0.770)	0.709 (0.657–0.757)
**Sensitivity**	**no obstruction**	0.825 (0.753–0.879)	0.824 (0.757–0.875)	0.873 (0.834–0.910)	0.774 (0.697–0.836)
	**partial obstruction**	0.824 (0.757–0.875)	0.763 (0.697–0.819)	0.731 (0.644–0.817)	0.730 (0.676–0.777)
	**complete obstruction**	0.900 (0.861–0.928)	0.855 (0.815–0.887)	0.678 (0.573–0.770)	0.709 (0.657–0.757)
**Specificity**	**no obstruction**	0.944 (0.92–0.962)	0.910 (0.884–0.931)	0.920 (0.880–0.955)	0.888 (0.861–0.911)
	**partial obstruction**	0.933 (0.906–0.952)	0.949 (0.927–0.965)	0.927 (0.898–0.954)	0.836 (0.798–0.867)
	**complete obstruction**	0.909 (0.871–0.937)	0.858 (0.817–0.891)	0.873 (0.838–0.905)	0.861 (0.826–0.891)

**F1-score**	**no obstruction**	0.819 (0.771–0.865)	0.771 (0.727–0.812)	0.906 (0.880–0.931)	0.682 (0.604–0.760)
	**partial obstruction**	0.819 (0.771–0.862))	0.800 (0.754–0.842)	0.729 (0.657–0.794)	0.737 (0.687–0.787)
	**complete obstruction**	0.906 (0.88–0.93)	0.861 (0.834–0.886)	0.604 (0.519–0.679)	0.746 (0.698–0.794)

Supplementary Table 2. Per-class performance metrics of the DISE-OTE-cause model for predicting the primary anatomical cause of airway obstruction. This table presents the accuracy, sensitivity, specificity, and F1-score for each class (oropharynx lateral wall, tongue base, and epiglottis) predicted using the DISE-OTE-cause model. The metrics are reported separately for the internal and external validation datasets.

Performance Metrics		OTE Obstruction Cause	
		**Internal Dataset**	**External Dataset**
**Accuracy**	**oropharynx lateral walls**	0.882 (0.797–0.935)	0.848 (0.811–0.890)
	**tongue base**	0.905 (0.830–0.949)	0.808 (0.750–0.864)
	**epiglottis**	0.878 (0.758–0.943)	0.783 (0.691–0.864)
**Sensitivity**	**oropharynx lateral walls**	0.882 (0.797–0.935)	0.848 (0.811–0.890)
	**tongue base**	0.905 (0.830–0.949)	0.808 (0.750–0.864)
	**epiglottis**	0.878 (0.758–0.943)	0.783 (0.691–0.864)
**Specificity**	**oropharynx lateral walls**	0.938 (0.885–0.967)	0.894 (0.860–0.927)
	**tongue base**	0.963 (0.916–0.984)	0.961 (0.940–0.979)
	**epiglottis**	0.939 (0.894–0.966)	0.885 (0.855–0.911)
**F1-score**	**oropharynx lateral walls**	0.888 (0.834–0.932)	0.865 (0.835–0.894)

	**tongue base**	0.925<br>(0.880–0.960)	0.859<br>(0.823–0.895)
	**epiglottis**	0.835<br>(0.750–0.901)	0.652<br>(0.573–0.723)

Supplementary Table 3. Comparison of our deep learning model with prior DISE scoring studies: prediction models and inter-rater agreement studies.

**Study**	**Purpose of the Study**	**Number of Participants**	**Performance Metrics (Cohen's kappa value)**	**Number of Scorers**	**External Validation**
Our study	Developing prediction model	1904	Obstruction degree<br>V: 0.78, OTE: 0.66<br>Obstruction cause (OTE): 0.83	N/A	✓
Hanif et al.	Developing prediction model	281	Obstruction degree<br>V: 0.55, OTE: 0.43<br>(O: 0.45, T: 0.38, E: 0.44)	N/A	✗
Koo et al.	Inter-rater variability	100	Obstruction degree<br>V: 0.52, OTE: 0.35<br>Obstruction cause (OTE): 0.25	62	N/A
Kezirian et al	Inter-rater variability	108	Obstruction degree<br>V: 0.60, OTE: 0.44<br>Obstruction cause (OTE): 0.86	2	N/A
Green et al.	Inter-rater variability	75	Obstruction degree<br>V: 0.40, OTE: 0.43<br>(O: 0.42, T: 0.60, E: 0.55)	4	N/A
Altintaş et al.	Inter-rater variability	55	Obstruction degree<br>V: 0.46, OTE: 0.41<br>(O: 0.37, T: 0.41, E: 0.45)	3	N/A

Supplementary Table 4. Summary of training hyperparameters and reproducibility information.

**Parameter**	**Value**
Learning rate	2×10^{-5} (2e-5)
Batch size	4
Optimizer	NAdam
Loss function	Cross-Entropy
Dropout rate	0.2
Attention setting	128-dim → 1 attention head (softmax pooling)
Training epochs	300
Training time	~ 10 hours per fold
Inference speed	~ 2 minutes per evaluation
